# Systematic Review of Economic Evaluation of Laparotomy versus Laparoscopy for Patients Submitted to Roux-en-Y Gastric Bypass

**DOI:** 10.1371/journal.pone.0099976

**Published:** 2014-06-19

**Authors:** Samanta Pereira Sussenbach, Everton Nunes Silva, Milene Amarante Pufal, Daniela Shan Casagrande, Alexandre Vontobel Padoin, Cláudio Corá Mottin

**Affiliations:** 1 Centro da Obesidade e Síndrome Metabólica do Hospital São Lucas, Pontifícia Universidade Católica do Rio Grande do Sul (COM HSL-PUCRS), Porto Alegre, Brasil; 2 Pós-Graduação em Medicina e Ciências da Saúde da Pontifícia Universidade Católica do Rio Grande do Sul, Porto Alegre, Brazil; 3 Faculdade de Ceilândia da Universidade de Brasília, Brasília, Brasil; 4 Postgraduate Program in Medical Sciences: Endocrinology and Metabolism, Universidade Federal do Rio Grande do Sul, Hospital de Clínicas de Port Alegre, Porto Alegre, Brazil; University of Utah School of Medicine, United States of America

## Abstract

**Background:**

Because of the high prevalence of obesity, there is a growing demand for bariatric surgery worldwide. The objective of this systematic review was to analyze the difference in relation to cost-effectiveness of access route by laparoscopy versus laparotomy of Roux en-Y gastric bypass (RYGB).

**Methods:**

A systematic review was conducted in the electronic databases MEDLINE, Embase, Scopus, Cochrane and Lilacs in order to identify economic evaluation studies that compare the cost-effectiveness of laparoscopic and laparotomic routes in RYGB.

**Results:**

In a total of 494 articles, only 6 fulfilled the eligibility criteria. All studies were published between 2001 and 2008 in the United States (USA). Three studies fulfilled less than half of the items that evaluated the results quality; two satisfied 5 of the required items, and only 1 study fulfilled 7 of 10 items. The economic evaluation of studies alternated between cost-effectiveness and cost-consequence. Five studies considered the surgery by laparoscopy the dominant strategy, because it showed greater clinical benefit (less probability of post-surgical complications, less hospitalization time) and lower total cost.

**Conclusion:**

This review indicates that laparoscopy is a safe and well-tolerated technique, despite the costs of surgery being higher when compared with laparotomy. However, the additional costs are compensated by the lower probability of complications after surgery and, consequently, avoiding their costs.

## Introduction

Obesity is a public health problem [1], [2], [3] that affects 1.7 billion people in the world [1]. It is considered a chronic disease of high prevalence and of difficult management, where it is often associated with important comorbidities such as Type 2 Diabetes Mellitus (T2DM), hypertension, dyslipidemias, and apnea [1], [4] which reduce life expectancy by 5 to 20 years and demands around 10% of health costs [5].

Roux en-Y gastric bypass (RYGB) surgery is considered the gold standard [6] for the surgical treatment of obesity by providing lower morbi-mortality to the patient [5], [7], [8] efficacious results [9], [10] and control of comorbidities [5], [7], [8].

Initially, bariatric surgery was only performed by laparotomy, and then RYGB began to be performed also by the laparoscopic route as described by Wittgrove et al. [11] in 1994. In the study performed by Buchwald and Oien (2013), the overall number of procedures performed in 2011 was presented; it was observed that U.S./Canada and Brazil lead the global surgical numbers, presenting 101.645 and 65,000 cases, respectively. In Brazil, from the total number of surgeries performed, only 6000 are held in the Brazilian public health system, which is performed by laparotomy, because there is no incorporation of laparoscopic procedures in the public health system. No specific data on the proportion between laparoscopy and laparotomy in Brazil and in the USA was found. Given that both of these routes shows a different set of costs and effects in health, it is necessary to analyze them together for the purpose of guiding decision making in their incorporation in health systems, by means of economic evaluations (cost-effectiveness and its variations).

The objective of this systematic review was to determine the difference in access route, laparoscopy versus laparotomy, for RYGB surgery in relation to cost- effectiveness.

## Methods

### Study Eligibility Criteria

Studies eligible for inclusion met the following criteria: (1) they presented economic evaluation (cost-effectiveness, cost-utility, cost-benefit, cost-minimization and cost-consequence), (2) they compared the surgical access routes (laparoscopic and laparotomic) for RYGB, (3) they evaluated adult patients (18 to 60 years of age), of both genders, (4) they evaluated patients who had class II obesity [body mass index (BMI) ≥35 kg/m^2^] with comorbidities, and class III obesity (BMI ≥40 kg/m^2^).

The studies excluded were those that did not demonstrate a direct comparison between the access routes; techniques that were not RYGB; obese patients who were not operated; literature review or letter to the editor; and studies that did not make an economic evaluation.

### Information Sources and Search Strategy

The search in electronic databases was performed through April 2012. The databases utilized were MEDLINE (via PubMed), Embase, Scopus, Cochrane and Lilacs, using the following terms: bariatric surgery/bariatrics/gastric bypass/anastomosis Roux-en-Y/costs and cost analysis/economics/cost-benefit analysis/health care costs/hospital costs/employer health costs/cost of illness/economics medical/biomedical technology/laparotomy/laparoscopy/and hand-assisted laparoscopy. We did not use any restriction in dates or languages when conducting the search. The whole search strategy performed for MEDLINE (via PubMed) can be seen in Appendix S1. This search strategy was adapted for the other databases.

### Study Selection and Data Extraction

This study was carried according to PRISMA [12] (Preferred Reporting Items for Systematic Reviews and Meta-analyses), standard for reporting systematic reviews and meta-analyses. Two reviewers (SPS and ENS), independently extracted the data according to a standardized form for the extraction of articles. Disagreements were resolved by consensus; evaluation by a third reviewer was not necessary. The data extracted were the following: country, year in which the cost was evaluated, currency, type of economic evaluation (cost-effectiveness; cost-utility; cost-benefit; cost-minimization; cost-consequence), perspective (society; public health; third player; hospital), population of patients, costs (direct; indirect; intangible), health outcomes (quality of life; mortality, pulmonary complications - pulmonary embolism, pneumonia, thrombosis-, cardiovascular complications, sepsis, incisional hernia, surgical wound infection, gastrointestinal hemorrhage, obstruction, anastomosis, intra-abdominal abscess, fistula, perforation, leak, weight loss, reintervention, hospitalization time, loss of blood).

### Quality Assessment

Drummond’s checklist [13] of 10 items was utilized to determine if the method in each study was methodologically adequate for proposed objectives and if the results were valid. It was adopted as the quality scale the following cutoffs: high-quality study (between 8 and 10 filled items); medium-high quality (entre 6 e7 filled items); medium-low quality study (between 4 and 5 filled items) e low-quality (4 below filled items).

Drummond’s checklist allows a systematic evaluation, whose points to be evaluated are discussed as follows: 1) definition of the research question; 2) comprehensive description of alternatives; 3) evidence of effectiveness; 4) relevance of costs and consequences; 5) measured accuracy of costs and consequences; 6) credibility of values of costs and consequences; 7) temporal adjustment of costs and consequences; 8) utilization of incremental analysis of costs and consequences of alternatives; 9) sensitivity analysis; 10) adequate discussion (based on index or calculation; comparison of results with other similar studies; discussion of generalization of results; evaluation of factors; and questions of implementation).

## Results

### Search Findings and Study Inclusion

**Figure 1:**
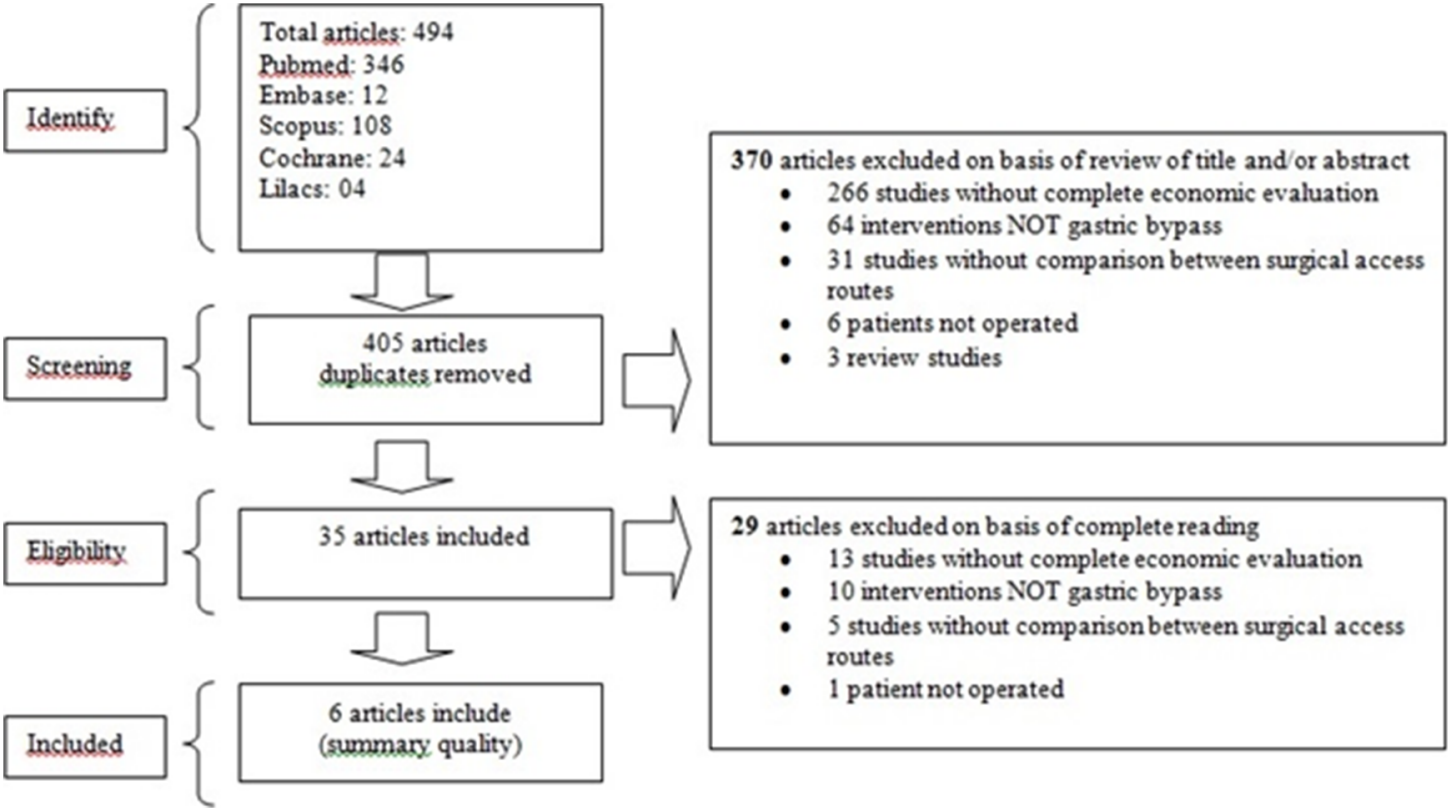
Flowchart.

The literature search identified 494 potentially relevant studies (Figure 1); 346 were from PubMed, 12 from Embase, 108 from Scopus, 24 from Cochrane and 4 from Lilacs. Among these, 89 were duplicates. From the 405 articles, 370 were excluded after reading the title and abstract. The remaining 29 articles were excluded on basis of complete reading. Therefore, 6 studies met the eligibility criteria.

### Studies Quality

Three studies [14], [15], [16] did not fulfill at least half of the items that evaluate the data quality. Two studies [17], [18] satisfied 5 of the required items, which demonstrate a medium-low quality, and only 1 study [8] fulfilled 7 of the 10 items, thereby considered a medium-high quality of information. One piece of essential information that was not included in any of the 6 studies was incremental analysis (Table 1).

**Table 1:** General characteristics of the studies selected.

Study	Well definedquestion	Adequatealternativesdescription	Evidence ofeffectiveness	Relevantcosts/consequences	Costs/consequencesmeasuredaccurately	Valuation costs,consequencescredible	Discounting usedas credible	Incrementalanalysesappropriatelyreported	Sensitivityanalysesreported	Adequatediscussion	N of itemsmet
Siddiqui2006	Yes	Yes	Yes(review ofthe literature)	Cannot tell (study’sPerspective is notinformed)	Cannot tell(study’s perspectiveis not informed)	Cannot tell(study’s Perspectiveis not informed)	Not applicable	No	Yes	Yes	7
Paxton2005	Yes	Yes	Yes(review ofthe literature)	Cannot tell (timehorizon and study’sperspective arenot informed)	Cannot tell(time horizonand study’sperspective arenot informed)	Cannot tell(time horizonand study’sperspective arenot informed)	Cannot tell(time horizon isnot informed)	No	No	Yes	5
Jones2006	Yes	Yes	Yes(retrospective studybased on review ofthe literaturewith morethan 25thousand patients)	No	Cannot tell(time horizonand study’sperspective arenot informed)	Cannot tell(time horizonand study’sperspective arenot informed)	Cannot tell(time horizon isnot informed)	No	No	No	3
Weller2008	Yes	Yes	Yes(national dataon hospitalstay withmore than 19thousand patients)	No(only hospitalcosts)	Yes	Yes(based onhospital records)	No	No	No	No	5
Nguyen2001	Yes	Yes	Yes(randomizedcontrolled trial)	Cannot tell(time horizonand study’sperspective arenot informed)	Cannot tell(time horizonand study’sperspective arenot informed)	No	Cannot tell(time horizon isnot informed)	No	No	No	3
Nguyen2002	Yes	Yes	Yes(review of theliterature)	Cannot tell(time horizonand study’sperspectiveare not informed)	Cannot tell(time horizonand study’sperspective arenot informed)	No	Cannot tell(time horizon isnot informed)	No	No	No	3

Source: Preparedbytheauthorsfromtheselectedstudies.

All economic evaluations were conducted in the United States (n = 6, one in 2002, another in 2004, two in 2005, and two [14], [15] did not provide this information), where the dollar was the currency utilized. The perspectives adopted in the studies were those of society [17] and the hospital [14], [18]; the others [8], [15], [16] did not provide this data.

All studies showed their respective type of economic evaluation, which alternated between cost-effectiveness and cost-consequence. The target population of all studies was patients subjected to RYGB surgery by the laparoscopic and laparotomic routes (Table 2).

**Table 2:** Critical evaluation of the studies selected.

Study	Country	Cost Year	Currency	Type of economicevaluation	Perspective	Target population
Siddiqui, 2006	EUA	2002	US$	Cost-Effectiveness	Not informed	Patients submitted to RYGB**(laparotomic and laparoscopic routes)over a year. Patients withBMI* ranges:Group A–BMI* 35–49 Kg/m^2^;Group B–BMI* 50–60 Kg/m^2^;Group C–BM*I>60 Kg/m^2^.
Paxton, 2005	EUA	2004	US$	Cost-Effectiveness	Society	Patients submitted to RYGB**(laparotomic and laparoscopic routes)Patients stratified by gender.
Jones, 2006	USA	2005	US$	Cost-Consequence	Not informed	Patients submitted to RYGB**(laparotomic and laparoscopic routes).
Weller, 2008	USA	2005	US$	Cost-Consequence	Hospital	Obese adults(≥18 years old)whounder went RYGB**(laparotomic and laparoscopic routes).
Nguyen, 2001	USA	Not informed	US$	Cost-Consequence	Hospital	Patients submitted to RYGB**(laparotomic and laparoscopic routes)with BMI* 40–60 Kg/m^2^and 21–60 years old.
Nguyen, 2002	USA	Not informed	US$	Cost-Consequence	Not informed	Patients submitted to RYGB**(laparotomic and laparoscopic routes)with BMI* 40–60 Kg/m^2^.

Source: Prepared by the authors from the selected studies.

*BMI = Body Mass Index,

**RYGB = Roux-en-Y Gastric Bypass.

The studies reported the surgical procedure as its direct cost, except Weller, 2008 (Table 3). Two studies [8], [17] considered clinical complications as a direct cost, while 3 studies [14], [15], [18] included the medical-hospital costs. Only one study [17] considered the indirect costs, which were related to loss of revenue due to early death.

**Table 3:** Description of costs, health endpoints and results of studies.

Study	Costs	Health endpoints	ICER*	Studyconclusion	Sensitivityanalysis
Siddiqui,2006	Direct costs: surgical proceduresand immediate complications (fistula,anastomotic stenosis, pneumonia,pulmonary embolism, wound infection)and late complications (incisionalhernia, cholelithiasis and surgeryrevision).	Mortality and immediateand late complications(one year) from theliterature review. Complicationsof laparotomy – highermortality, pulmonaryembolism, pneumonia,incisional hernia, surgicalwound. Complications oflaparoscopy-gastrointestinalbleeding, obstruction andanastomosis.	No	Laparoscopy is a dominant strategy(greater health benefits and lower costs)compared to laparotomy,taking into account literature dataon mortality and complications,as well as cost data.The attractiveness of laparascopytends to be lower when the BMIincreases as there are moresurgical risks for patients with BMI>60.	Sensitivity analysis of univariate andmultivariate (3-way).Variables used: mortality rateand complications(immediate and late).Sensitivity analyzesconfirmed theresults of the cost-effectiveness:laparoscopy is prefer ableto laparotomy.
Paxton,2005	Direct and indirect costs.Indirect costs were includedsurgery, routine procedures,hospitalizations, complications(15 types of complications) andrisk of conversion fromlaparoscopy to laparotomy.In indirect costs wasincluded income loss for early death.	Mortality rate, complications andin come loss for early death,from literature review.Complications of laparotomy-thrombosis, pulmonary embolism,pneumonia, intra-abdominalabscess, fistula, wound infection,incisional hernia. Complications oflaparoscopy -anastomosis,perforation, gastrointestinalbleeding, obstruction.	No	Considering the complications andtheir probabilities of occurrencein each access route, the periodof hospitalization/recovery andmortality in laparoscopy proveddominant strategy(lower cost and greaterclinical benefit).	No
Jones,2006	Direct costs: time of surgery,length of stay in hospital,inputs associated to surgery.	Incidence leak, obstruction,wounds, weight loss,recovery time period from surgeryand mortality. Complications oflaparoscopy more likely toleak and obstruction.	No	Laparoscopy has direct costshigher and higher leak rateof obstruction and similarweight loss over the longterm. Thus, laparotomy wouldbe the preferred access route.	No
Weller,2008	Direct costs: medicaland hospital costs.	Occurrence of one or morecomplications (pulmonary andcardiovascular related to surgery),reoperation, mortality, duration ofhospitalization. Complications oflaparotomy-pulmonary complications(embolism, thrombosis),cardiovascular, sepsis, anastomotic,mortality, reintervention.	No	Patients undergoing laparoscopyare less likely of reinterventionand postoperative complications(cardiovascular, pulmonaryrelated to surgery, sepsis, and fistula),and shorter length ofstay in hospital. Totalcosts were similar betweenthe two access routes.	No
Nguyen,2001	Direct medical and hospital costs:surgical procedures,medical tests,hospitalization, medicineand monitoring; and non-medicalhospital (overhead costs).	Outcomes of quality of life(SF-36 and BAROS)and clinical effects(length of stay,blood loss, complications) fromrandomized clinical trial byintention to treat. Complicationsof laparotomy-embolism,obstruction, wound infection,fistula, anastomosis, hernia.	No	Laparoscopy proved to be morecost-effective compared tolaparotomy, given that there wasno significant difference betweenthe total costs of the interventions,in addition to having greaterhealth benefits (improvedquality of life, shorter hospitalstay, faster recovery andshorter period return to thelabor market). Regardingcomplications, there was nosignificant difference between groups.	No
Nguyen,2002	Direct medical and hospitalcosts: surgical procedures,medical tests,hospitalization,medicines and monitoring;and non-medicalhospital (overhead costs).	Outcomes of quality of life(SF-36 and BAROS) andclinical effects (length of stay,blood loss, complications).Complications of laparotomy-anastomosis,wound infection, obstruction,anastomotic, bleeding, thrombosis.	No	Laparoscopy is safe andeffective compared to laparotomy,with lower rates of mortality,complications, recovery time andreturn to the labor market. Theincremental costs of laparoscopytend to be offset by incrementalbenefits (clinical and quality of life)compared to laparotomy.	No

Source: Prepared by the authors from the selected studies.

*ICER, incremental cost-effectiveness ratio.

All studies, except that of Nguyen, 2002, observed that medium hospitalization time was between 2 and 3 days, which was lesser than in patients that underwent surgery by the laparoscopic route (between 3 and 4 days) (Table 4).

**Table 4:** Mortality, complications, surgical cost, days of hospitalization and return to labor market of the studies selected.

Studies	Mortality		Complications						Surgical cost		Days of hospitalization		Returnto labor market	
			Fistula anastomosis		Incisional hernia		Gastrointestinal obstruction							
	LGB*	OGB**	LGB*	OGB**	LGB*	OGB**	LGB*	OGB**	LGB*	OGB**	LGB*	OGB**	LGB*	OGB**
Siddiqui 2006	0.2%	0.9%	2.0%	1.7%	0.5%	8.6%	1.8%	0%	U$ 5,830	U$ 4,304	2,5 days	3,7 days	NR***	NR***
Paxton 2005	0.4%	0.6%	1.9%	1.7%	0.4%	2.9%	2.6%	1.0%	U$ 5,830	U$ 4,304	2,5 days	3,7 days	NR***	NR***
Jones 2006	0.2%	0.2%	2.0%	0.4%	0.3%	6.6%	>3%	0.4%	NR***	NR***	2,5 days	3,4 days	NR***	17 days
Weller 2008	0.1%	0.3%	1.4%	2.0%	NR***	NR***	NR***	NR***	NR***	NR***	2,0 days	3,0 days	NR***	NR***
Nguyen 2001	NR***	NR***	1.3%	1.3%	0%	7.9%	1.3%	0%	U$ 4,922¥	U$ 3,591¥	3,0 days	4,0 days	32,2 days	46,1 days
Nguyen 2002	0%	0%	1.3%	2.6%	NR***	NR***	5.1%	0%	NR***	NR***	NR***	NR***	32,2 days	46,1 days

Source: Prepared by the authors from the selected studies.

*LGB = Laparoscopic Gastric Bypass,

**OGB = Open Gastric Bypass or Laparotomy,

***NR = Not Reported,

¥Average of surgical cost. (U$ 4,922.00±1,927.00 - laparoscopy and U$ 3,591.00±1,000.00 - laparotomy)

The learning curve of the laparoscopic route is referenced in the majority of studies [8], [15], [16], [17] according to Siddiqui, 2006, laparoscopy is a technically challenging procedure, and it is associated with longer surgical time and higher rates of perioperative complications. Nguyen, 2002, observed that laparoscopy requires that the surgeon has overcome the steep learning curve of the complex laparoscopic procedure. Paxton, 2005, also suggested that the well-trained surgeons involved need a learning curve of 50–200 surgeries by laparoscopy before refining their laparoscopic technique.

In the description of health outcomes, five studies [8], [15], [16], [17], [18] reported rate of mortality; by the laparotomic route it varied between 0 and 0.87%, and the laparoscopic between 0 and 0.36% (Table 4). All studies showed surgical complications with both laparoscopic and laparotomic routes [8], [14], [15], [16], [17], [18]; with respect to incidence of fistula in the postoperative period, there was no consensus between the studies in relation to which route was more recurrent. Regarding the presence of incisional hernia, two studies [15], [18] did not report this aspect, and the others reported greater incidence with laparotomic route (variation of 2.89 to 8.58%) than with the laparoscopic route (variation of 0 to 0.47%). Regarding to intestinal obstruction, one study [18] did not report this aspect, but the others noted greater incidence in the laparoscopic route (variation of 1.26 to 5.1%) in relation to laparotomy (variation of 0 to 1.05%) (Table 4).

No study showed a cost-benefit ratio, also known as incremental ratio. Only one study [8] showed deterministic sensitivity analysis (uni- and multivariate), which evaluated the mortality rate and immediate and late complications. This analysis confirmed that laparoscopy is preferable to laparotomy.

Five studies [8], [14], [15], [17], [18] considered the surgery by laparoscopy the dominant strategy because it showed greater clinical benefit (Table 3) and lower total cost after one year (Table 1), despite the surgical cost being higher at the beginning (Table 4). Besides, lower occurrence of mortality was reported [8], [15], [17] (Tables 3,4), less probability of complications [8], [15], [18] (Table 3,4) and shorter return to work time [14], [15] in the laparoscopic group (Table 4).

## Discussion

Three studies were rated as medium quality compared to the Drummond checklist (Drummond et al., 2005). One was classified as medium-high quality (Siddiqui) and two medium-low quality (Weller and Paxton). Three studies were considered low quality, because they fulfilled just three out of the ten proposed items in the checklist. As highlighted by Drummond (2005) “it is unrealistic to expect every study to satisfy all of the points; However, the systematic application of these points will allow readers to identify and assess the strengths and weakness of individual studies”.

Dealing with qualitative results, laparoscopy was shown in the majority of studies [8], [14], [15], [17], [18] as being preferred over laparotomy, since the additional cost of laparoscopy would be compensated by the lower probability of the occurrence of complications of the surgery, shorter time to return to the work force and greater clinical benefit. Thus, this route can be considered a dominant strategy, as concluded in two studies [8], [17]. When analyzing just those studies that met at least half of the items proposed by the checklist, all [8], [17], [18] considered laparoscopy as the superior strategy.

These lines of clinical evidence favoring laparoscopy are corroborated by studies that reinforce the lower mortality [19], [20] and reduced morbidity [19], [21], besides the more rapid recovery in the postoperative period [22], such as better healing [23], reduction of immediate and late complications, such as, respectively, the surgical wound infection [22], [23] and incisional hernias, fistulas and adherences [24]. According to Reosch, et al. [22], the patient who undergoes surgery by laparoscopy shows 79% less chance of infection in the surgical wound and 89% fewer complications such as hernia, when compared with patients submitted to laparotomy. However, Podnos et al. [20] reports that up to 20% of patients show incisional hernias. Studies [19], [22], [23] reinforce that there is a shorter hospitalization period.

However, surgery by laparoscopy requires a substantial learning curve so that a surgeon develops technical ability for a successful operation; his experience and training in the technique will produce better surgical results [25], [26]. According to El-Kadre, 2013, the relative risk of postoperative complications, mortality and conversion decreases with increased experience of the surgery team, and tends to stabilize after a learning curve of 500 procedures, although the surgeon individual learning process occurs at different speeds. The exact number of surgeries is arguable, because it is related to the surgeon experience with laparoscopic suturing and stitching ability, and with his operatory experience with laparotomy and other advanced laparoscopic surgeries [15].

According to Podnos, 2003, the incidence of postoperative wound infection in laparoscopic surgery is approximately 2.9%, while surgery by laparotomy shows a rate of 6.6%. In the laparotomic route, there is more often iatrogenic splenectomy and complications in the abdominal wall, besides the return to normal activities being slower. However, 2.25% of surgeries started by laparoscopy need to be converted to laparotomy, due to hepatomegaly (47.5%) and excess intra-abdominal fat (23.8%) [17].

Bariatric surgery is a procedure that has high costs [27], but it is effective in resolving comorbidities and the loss of weight, when compared to the conservative treatment of obesity [28]. Nguyen et al [14] found that the costs of laparoscopy were 37% higher due to the duration of the surgery and to the instruments not being reusable, but that the operational costs of laparoscopy were compensated by 33% reduction in hospital services, since there was less hospitalization time, reflecting the reduction in utilizing nursing services, diagnostic services, etc. According to Faria et al. [5] in comparing the best medical treatment for obesity with the surgical technique of gastric bypass in the global population of patients with BMI>35 kg/m^2^, the procedure generates a saving of approximately 13,244€ per patient. The benefits in terms of cost- effectiveness are superior for those patients who are younger, have a BMI between 40 and 50 kg/m^2^, and do not have comorbidities related to obesity. Some studies [29], [30] estimate that the surgery would have its expense compensated over the years just considering the savings on medications. However, these studies followed the patients for a short period of time. McEwen et al [31] reports that the medical costs increase in the 6 months preceding the surgery, keeping high in the beginning of the first post-surgical year, due to the expenses for drugs and diagnostic tests, but tend to decline in the course of the year after the surgery. However, Neovious et al. [9] believes that bariatric surgery is associated with an increased use of health services in the first 6 years after the surgery. Since there is no consistency in the information in the literature with regard to the durability of outcome and diminution of expenses, there is a need for further studies with long-term follow-up of patients submitted to bariatric surgery.

In this systematic review, the study by Jones et al. [16] was the only one that showed divergent data regarding the preference of surgical access of RYGB, due to the greater probability of leak and obstruction in laparotomy surgery. Besides, the authors did not observe a significant difference in weight loss and considered that the laparoscopic route was associated with higher costs. However, some points should be emphasized: (1) the type of study adopted was cost-consequence, which is methodologically inferior to cost-effectiveness analysis, because it does not meet all the necessary criteria, even taking into consideration a large sample (25,000 patients submitted to bariatric surgery); (2) in economic evaluation, it is not necessary for the intervention to be cost-saving, depending on the willingness to pay of the decision maker (cost-effectiveness threshold); (3) the critical evaluation showed that the study fulfilled 30% of the items proposed on Drummond’s checklist, indicating that the evidence generated shows significant methodological faults.

Nowadays, the cost-effectiveness of laparoscopy versus laparotomy does not seem to be a priority issue, since the last study regarding this topic was published in 2008. The probable reason for this is that most health systems have adopted laparoscopy in their medical routine. However, some countries such as Brazil have not introduced laparoscopy in the public health system yet. In Brazil, for example, the public system is responsible for 75% of medical care [32] and treats approximately three million morbidly obese patients [33].a Due to lack of information in the literature, the costs included in this systematic review represent knowledge and learning curve for the period 2001–2008. Since then, surgeons have improved their skills in conducting laparoscopy, which tend to reflect in fewer complications and, therefore, less costs. Unfortunately, this hypothesis could not be tested or proven from the studies analyzed in this systematic review because the long period of times does not allow the comparison of costs.

Our results may suggest the benefits of laparoscopy over laparotomy. Moreover, these findings may also contribute to the empirical knowledge, since this is the first study to apply the method of systematic review of economic evaluation related to bariatric surgery, which contribute to inform and consolidate information effects on health and costs.

## Conclusion

We conclude that laparoscopy has been demonstrated to be safe and well-tolerated technique, despite the costs of the surgery being higher when compared with laparotomy. However, the additional costs are compensated by the lower probability of complications after the surgery and, consequently, avoiding the costs to reverse them. Since economic evaluations take into consideration the dimension of costs, besides effects on health, it is emphasized that the extrapolation of these results to other contexts – countries with different costs structure – cannot be determined, because all studies analyzed in this systematic review were from the USA. Thus, it is necessary to conduct studies in other contexts, to confirm this pattern. We believe that this study can help in decision making in countries where laparoscopy is not available, such as in the case of Brazil, where it is only established in the private sector.

This paper is the first application of the method of systematic review to economic assessment studies on bariatric surgery. This tool has been recognized worldwide with the best way to critically summarize health effects and costs of competing technologies in health.

## Supporting Information

Appendix S1 **Search strategy of in the whole study conducted using MEDLINE (via PubMed).**
(DOCX)

Checklist S1 **PRISMA 2009 checklist.**
(DOC)
