# Supplementary material for: Systematic Review of Economic Evaluation of Laparotomy versus Laparoscopy for Patients Submitted to Roux-en-Y Gastric Bypass
Source: PLoS One. 2014 Jun 19;9(6):e99976. doi: 10.1371/journal.pone.0099976 (PMC4063755; doi:10.1371/journal.pone.0099976)
Supplement: Appendix S1 — Search strategy of in the whole study conducted using MEDLINE (via PubMed). (DOCX) [file pone.0099976.s001.docx]

Appendix 1 – Search strategy in the whole study conducted using MEDLINE (via PubMed)

("Bariatric Surgeries"[tw] OR "Bariatric surgery"[MeSH Terms] OR “Bariatrics”[MeSH Terms] OR "Gastric Bypass"[MeSH Terms] OR "Bypass, Gastric"[tw] OR "Roux-en-Y Gastric Bypass"[tw] OR "Gastric Bypass, Roux-en-Y"[tw] OR "Roux en Y Gastric Bypass"[tw] OR "Greenville Gastric Bypass"[tw] OR "Gastroileal Bypass"[tw] OR "Gastrojejunostomy"[tw] OR "Gastrojejunostomies"[tw] OR "Anastomosis, Roux-en-Y"[MeSH Terms] OR "Anastomosis, Roux en Y"[tw] OR "Roux-en-Y Loop"[tw] OR "Roux en Y Loop"[tw] OR "Roux-en-Y Loops"[tw] OR "Roux-en-Y Anastomosis"[tw] OR "Roux en Y Anastomosis"[tw] OR "Roux-en-Y Anastomoses"[tw] OR "Roux-en-Y Diversion"[tw] OR "Roux en Y Diversion"[tw] OR "Roux-en-Y Diversions"[tw] OR "Bariatric Medicine"[Mesh] AND ("Costs and Cost Analysis"[Mesh] OR "Economics"[Mesh] OR "economics" [Subheading] OR "Cost-Benefit Analysis"[Mesh] OR "Health Care Costs"[Mesh] OR "Hospital Costs"[Mesh] OR "Employer Health Costs"[Mesh] OR "Cost of Illness"[Mesh] OR "Cost*"[tw] OR "Cost-effectiveness"[tw] OR "Cost-utility"[tw] OR "Cost-benefit"[tw] OR "Cost-minimization"[tw] OR "Cost-consequence"[tw] OR "economic evaluation"[tw] OR "Economics, Medical"[Mesh] OR "Biomedical Technology"[Mesh] OR "health technology assessment"[tw] AND ("Laparotomy"[Mesh] OR "laparotomy economics"[tw] OR "Laparotomy methods"[tw] OR "Laparotomies"[tw] OR "conventional bariatric surgery"[tw] OR "conventional bariatric"[tw] OR "open surgery"[tw] OR "open gastric bypass"[tw] OR "Laparoscopy"[Mesh] OR "Hand-Assisted Laparoscopy"[Mesh] OR "Laparoscopic surgery"[tw] OR "Laparoscopic gastrectomy"[tw] OR "Robotic-assisted laparoscopic"[tw] OR "Laparoscopic bariatric surgery" OR "Digestive System Surgical Procedures"[Mesh] OR "Laparoscopic bariatric"[tw] OR "Laparoscop*"[tw])
