# Supplementary material for: Systematic Review of Economic Evaluation of Laparotomy versus Laparoscopy for Patients Submitted to Roux-en-Y Gastric Bypass
Source: PLoS One. 2014 Jun 19;9(6):e99976. doi: 10.1371/journal.pone.0099976 (PMC4063755; doi:10.1371/journal.pone.0099976)
Supplement: Checklist S1 — PRISMA 2009 checklist. (DOC) [file pone.0099976.s002.doc]

| **Section/topic** | **#** | **Checklist item** | **Reported on page #** |
| --- | --- | --- | --- |
| **TITLE** | | |  |
| Title | 1 | **SYSTEMATIC REVIEW OF ECONOMIC EVALUATION OF LAPAROTOMY VERSUS LAPAROSCOPY FOR PATIENTS SUBMITTED TO ROUX-EN-Y GASTRIC BYPASS** | First page |
| **ABSTRACT** | | |  |
| Structured summary | 2 | **Background:** Because of the high prevalence of obesity, there is a growing demand for bariatric surgery worldwide. The objective of this systematic review was to analyze the difference in relation to cost-effectiveness of access route by laparoscopy versus laparotomy of Roux en-Y gastric bypass (RYGB).  **Methods:** A systematic review was conducted in the electronic databases MEDLINE, Embase, Scopus, Cochrane and Lilacs in order to identify economic evaluation studies that compare the cost-effectiveness of laparoscopic and laparotomic routes in RYGB.  **Results:** In a total of 494 articles, only 6 fulfilled the eligibility criteria. All studies were published between 2001 and 2008 in the United States (USA). Three studies fulfilled less than half of the items that evaluated the results quality; two satisfied 5 of the required items, and only 1 study fulfilled 7 of 10 items. The economic evaluation of studies alternated between cost-effectiveness and cost-consequence. Five studies considered the surgery by laparoscopy the dominant strategy, because it showed greater clinical benefit (less probability of post-surgical complications, less hospitalization time) and lower total cost.  **Conclusion:** This review indicates that laparoscopy is a safe and well-tolerated technique, despite the costs of surgery being higher when compared with laparotomy. However, the additional costs are compensated by the lower probability of complications after surgery and, consequently, avoiding their costs. | abstract |
| **INTRODUCTION** | | |  |
| Rationale | 3 | The objective of this systematic review was to determine the difference in access route, laparoscopy versus laparotomy, for RYGB surgery in relation to cost- effectiveness. | introduction |
| Objectives | 4 | P – patient; I – laparotomy and laparoscopy; C – no; O – no; S – economic evaluation | Methods |
| **METHODS** | | |  |
| Protocol and registration | 5 | We dont’t have protocol. |  |
| Eligibility criteria | 6 | Studies eligible for inclusion met the following criteria: (1) they presented economic evaluation (cost-effectiveness, cost-utility, cost-benefit, cost-minimization and cost-consequence), (2) they compared the surgical access routes (laparoscopic and laparotomic) for RYGB, (3) they evaluated adult patients (18 to 60 years of age), of both genders, (4) they evaluated patients who had class II obesity [body mass index (BMI) ≥ 35 kg/m^2^] with comorbidities, and class III obesity (BMI ≥ 40 kg/m^2^). | Methods |
| Information sources | 7 | The search in electronic databases was performed through April 2012. The databases utilized were MEDLINE (via PubMed), Embase, Scopus, Cochrane and Lilacs. | Methods |
| Search | 8 | bariatric surgery / bariatrics / gastric bypass / anastomosis Roux-en-Y / costs and cost analysis / economics / cost-benefit analysis / health care costs / hospital costs / employer health costs / cost of illness / economics medical / biomedical technology / laparotomy / laparoscopy / and hand-assisted laparoscopy | Methods |
| Study selection | 9 | Studies eligible for inclusion met the following criteria: (1) they presented economic evaluation (cost-effectiveness, cost-utility, cost-benefit, cost-minimization and cost-consequence), (2) they compared the surgical access routes (laparoscopic and laparotomic) for RYGB, (3) they evaluated adult patients (18 to 60 years of age), of both genders, (4) they evaluated patients who had class II obesity [body mass index (BMI) ≥ 35 kg/m^2^] with comorbidities, and class III obesity (BMI ≥ 40 kg/m^2^). | Methods |
| Data collection process | 10 | The data extracted were the following: country, year in which the cost was evaluated, currency, type of economic evaluation (cost-effectiveness; cost-utility; cost-benefit; cost-minimization; cost-consequence), perspective (society; public health; third player; hospital), population of patients, costs (direct; indirect; intangible), health outcomes (quality of life; mortality, pulmonary complications - pulmonary embolism, pneumonia, thrombosis-, cardiovascular complications, sepsis, incisional hernia, surgical wound infection, gastrointestinal hemorrhage, obstruction, anastomosis, intra-abdominal abscess, fistula, perforation, leak, weight loss, reintervention, hospitalization time, loss of blood). | Methods |
| Data items | 11 | The search in electronic databases was performed through April 2012 | Methods |
| Risk of bias in individual studies | 12 | The studies excluded were those that did not demonstrate a direct comparison between the access routes; techniques that were not RYGB; obese patients who were not operated; literature review or letter to the editor; and studies that did not make an economic evaluation. | Methods |
| Summary measures | 13 | We dont’t have. |  |
| Synthesis of results | 14 | We dont’t have. |  |

Page 1 of 2

| **Section/topic** | **#** | **Checklist item** | **Reported on page #** |
| --- | --- | --- | --- |
| Risk of bias across studies | 15 | We dont’t have. |  |
| Additional analyses | 16 | We dont’t have. |  |
| **RESULTS** | | |  |
| Study selection | 17 | The literature search identified 494 potentially relevant studies (Figure 1); 346 were from PubMed, 12 from Embase, 108 from Scopus, 24 from Cochrane and 4 from Lilacs. Among these, 89 were duplicates. From the 405 articles, 370 were excluded after reading the title and abstract. The remaining 29 articles were excluded on basis of complete reading. Therefore, 6 studies met the eligibility criteria. | Results |
| Study characteristics | 18 | Economic Evaluation | Methods |
| Risk of bias within studies | 19 | No |  |
| Results of individual studies | 20 | No |  |
| Synthesis of results | 21 | Three studies did not fulfill at least half of the items that evaluate the data quality. Two studies satisfied 5 of the required items, which demonstrate a medium-low quality, and only 1 study fulfilled 7 of the 10 items, thereby considered a medium-high quality of information. One piece of essential information that was not included in any of the 6 studies was incremental analysis | Results |
| Risk of bias across studies | 22 | No |  |
| Additional analysis | 23 | No |  |
| **DISCUSSION** | | |  |
| Summary of evidence | 24 | This analysis confirmed that laparoscopy is preferable to laparotomy. | Discussion |
| Limitations | 25 | Nowadays, the cost-effectiveness of laparoscopy versus laparotomy does not seem to be a priority issue, since the last study regarding this topic was published in 2008. The probable reason for this is that most health systems have adopted laparoscopy in their medical routine. However, some countries such as Brazil have not introduced laparoscopy in the public health system yet. In Brazil, for example, the public system is responsible for 75% of medical care and treats approximately three million morbidly obese patients. Due to lack of information in the literature, the costs included in this systematic review represent knowledge and learning curve for the period 2001-2008. Since then, surgeons have improved their skills in conducting  laparoscopy, which tend to reflect in fewer complications and, therefore, less costs. Unfortunately, this hypothesis could not be tested or proven from the studies analyzed in this systematic review because the long period of times does not allow the comparison of costs. | Discussion |
| Conclusions | 26 | Our results may suggest the benefits of laparoscopy over laparotomy. Moreover, these findings may also contribute to the empirical knowledge, since this is the first study to apply the method of systematic review of economic evaluation related to bariatric surgery, which contribute to inform and consolidate information effects on health and costs. | Conclusion |
| **FUNDING** | | |  |
| Funding | 27 | There is no financial interest by any of the authors. This study was supported by the Brazilian Government (FINEP). "The funders had no role in study design, data collection and analysis, decision to publish, or preparation of the manuscript". |  |

*From:*  Moher D, Liberati A, Tetzlaff J, Altman DG, The PRISMA Group (2009). Preferred Reporting Items for Systematic Reviews and Meta-Analyses: The PRISMA Statement. PLoS Med 6(6): e1000097. doi:10.1371/journal.pmed1000097

For more information, visit: **www.prisma-statement.org**.

Page 2 of 2
